# A decision-theoretic approach to the evaluation of machine learning algorithms in computational drug discovery

**DOI:** 10.1093/bioinformatics/btz293

**Published:** 2019-05-09

**Authors:** Oliver P Watson, Isidro Cortes-Ciriano, Aimee R Taylor, James A Watson

**Affiliations:** 1 Goring on Thames, Evariste Technologies Ltd., RG8 9AL UK; 2 Department of Chemistry, Centre for Molecular Science Informatics, University of Cambridge, Lensfield Road, Cambridge CB2 1EW, UK; 3 Department of Epidemiology, Center for Communicable Disease Dynamics, Harvard T.H. Chan School of Public Health, Boston, MA 02115 USA; 4 Infectious Disease Microbiome Program, Broad Institute, Cambridge, MA 02142 USA; 5 Nuffield Department of Medicine, Centre for Tropical Medicine and Global Health, University of Oxford, Oxford OX3, 7LF UK; 6 Mahidol-Oxford Tropical Medicine Research Unit, Faculty of Tropical Medicine, Mahidol University, Bangkok 10400, Thailand

## Abstract

**Motivation:**

Artificial intelligence, trained via machine learning (e.g. neural nets, random forests) or computational statistical algorithms (e.g. support vector machines, ridge regression), holds much promise for the improvement of small-molecule drug discovery. However, small-molecule structure-activity data are high dimensional with low signal-to-noise ratios and proper validation of predictive methods is difficult. It is poorly understood which, if any, of the currently available machine learning algorithms will best predict new candidate drugs.

**Results:**

The quantile-activity bootstrap is proposed as a new model validation framework using quantile splits on the activity distribution function to construct training and testing sets. In addition, we propose two novel rank-based loss functions which penalize only the out-of-sample predicted ranks of high-activity molecules. The combination of these methods was used to assess the performance of neural nets, random forests, support vector machines (regression) and ridge regression applied to 25 diverse high-quality structure-activity datasets publicly available on ChEMBL. Model validation based on random partitioning of available data favours models that overfit and ‘memorize’ the training set, namely random forests and deep neural nets. Partitioning based on quantiles of the activity distribution correctly penalizes extrapolation of models onto structurally different molecules outside of the training data. Simpler, traditional statistical methods such as ridge regression can outperform state-of-the-art machine learning methods in this setting. In addition, our new rank-based loss functions give considerably different results from mean squared error highlighting the necessity to define model optimality with respect to the decision task at hand.

**Availability and implementation:**

All software and data are available as Jupyter notebooks found at https://github.com/owatson/QuantileBootstrap.

**Supplementary information:**

Supplementary data are available at *Bioinformatics* online.

## 1 Introduction

Empirical methodologies guide a significant proportion of early-stage small-molecule drug discovery (Cumming *et al.*, 2013; Keiser *et al.*, 2007; Lipinski, 2004). These range from simple rule-based methods (Lipinski’s rule of 5), to searching over molecules ‘similar’ to those already known, to using more complex regression models. This work concerns the objective evaluation of the predictive ability of the latter, namely statistical and machine learning regression models trained on molecular structure-activity data. The goal of these models is to characterize the relationship between a high-dimensional binary vector representation of small molecules (known as a molecular fingerprint) and the corresponding target specific *in vitro* activities. In this context, use of regression modelling is often known as quantitative structure-activity relationship modelling (QSAR) (Sliwoski *et al.*, 2014; Van De Waterbeemd and Gifford, 2003), and many different model classes have been used: support vector machines (Burbidge *et al.*, 2001), ridge regression (Nandi *et al.*, 2007), neural nets (Ajay *et al.*, 1998; Lenselink *et al.*, 2017; Nandi *et al.*, 2007; Sadowski and Kubinyi, 1998) and random forests (Svetnik *et al.*, 2003), to name but a few. The success of these models is in part due to high-throughput screening experiments which produce large structure-activity datasets (order of magnitude 10^2^–10^6^ datapoints).

Regression with high-dimensional bioinformatic data is known to be difficult. Problems include *the curse of dimensionality*, optimization bias, reporting bias and low signal-to-noise ratios (Boulesteix and Strobl, 2009; Castaldi *et al.*, 2011; Ioannidis, 2005; Jelizarow *et al.*, 2010; Matveeva *et al.*, 2016; Somorjai *et al.*, 2003; Zervakis *et al.*, 2009). A major theoretical framework underpinning the use and interpretation of computational methods for complex data modalities is cross-validation (Geisser, 1975; Stone, 1974), which provides an estimate of the predictive error rate (Castaldi *et al.*, 2011; Molinaro *et al.*, 2005). However, validation strategies based on random partitioning of datasets, either by *K*-fold cross-validation or the bootstrap, are known to be optimistic for structure-activity modelling (Sheridan, 2013; Wallach and Heifets, 2018; Wu *et al.*, 2018). Multiple alternative strategies have been proposed, for example, splitting by date of assay (Sheridan, 2013), constructing local neighbourhoods based on similarity scores or scaffold splitting (Sheridan, 2013; Wu *et al.*, 2018), or stratified sampling whereby equal distributions of the activity levels are assured across training and testing sets (Wu *et al.*, 2018). These alternative strategies can suffer from the same issues as standard cross-validation or rely on strong data assumptions. A better general approach is needed.

## 2 Approach

This work reiterates that standard validation approaches—*K*-fold cross-validation and the bootstrap—based on random partitioning of available data, will not target the true predictive model error in the context of small-molecule drug discovery. We give a theoretical justification for this claim and show it empirically using 25 publicly available datasets. We propose a simple alternative partitioning method—the quantile-activity bootstrap—which splits datasets on quantiles of the activity distribution function. This univariate parametrization of the training set construction allows for inference on the predictive ability of different regression methods in the limit: as information in the training set is reduced to almost zero. In addition, we argue that out-of-sample model performance should be evaluated from a decision-theoretic perspective (Savage, 1954) using loss functions, which reflect as best possible the process of drug discovery. Tailor-made loss functions will better determine truly optimal model classes compared with standard goodness-of-fit metrics. We propose simple rank-based loss functions to evaluate out-of-sample model prediction accuracy. We show that in these low signal-to-noise settings (Cortés-Ciriano and Bender, 2016; Kalliokoski *et al.*, 2013a, b; Kramer *et al.*, 2012), models with greater structural constraints (ridge regression and linear kernel support vector regression) outperform less constrained machine learning algorithms (neural nets and random forests).

## 3 Materials and Methods

### 3.1 Cross-validation with biased data

#### 3.1.1 Problem setting

This section outlines the formal framework and notation we use throughout the article. We consider the general problem of comparing the performance of multiple predictive models (statistical and machine learning) with respect to a given dataset. ‘Optimality’ of these predictive models is evaluated with respect to a subjective loss function.

The context investigated here is finding ‘active’ molecules within molecular space. ‘Active’ is defined as having activity level above a given threshold. This activity is target specific. Conditional on a given initial dataset, the overall loss (negative utility of the model) is defined as a function of the number of new molecules needed to be tested until an active molecule is reached.

Each molecule is represented by its ‘molecular fingerprint’, a *P*-dimensional binary vector. We denote this as $x_{i}={\left\{x_{i}^{j}\right\}}_{j=1}^{P}$, where *i* indexes the molecule and *j* indexes the feature (as referred to in the machine learning literature) or covariate (statistics literature). We have *P *=* *128 for the fingerprint representation used in this analysis. Each molecule *x_i_* has a target specific activity *y_i_* which corresponds to the negative logarithmic *in vitro* half-inhibitory concentration (p-IC_50_: higher values correspond to increased activity). In this section, we ignore the target specificity as each dataset has an associated target and the datasets are analysed independently. We do not consider multi-objective regression models here. We denote the (unknown) functional relationship between the fingerprint and the outcome (activity) as y=G(x)+ϵ, where *ϵ* is experimental error (general regression framework).

Given a choice of models $M_{1},\ldots ,M_{T}$, respective performances are commonly evaluated using *K*-fold cross-validation (Molinaro *et al.*, 2005; Stone, 1974) [detailed description given in Friedman *et al.* (2001), Chapter 7], or the bootstrap (Efron, 1983) which is closely related. Standard *K*-fold cross-validation proceeds by dividing the data ${\left\{(x_{i},y_{i})\right\}}_{i=1}^{N}$ into a partition of *K *>* *1 equally sized subsets $S_{1},\ldots ,S_{K}$. For the *k*th subset, we train (fit) our model *M_t_* using the data $S_{\text{train}}=\underset{m\neq k}{\cup }S_{m}$. The out-of-sample expected loss is then estimated by testing on elements in *S_k_*: $l_{k}=L\left[{\left\{y_{i}\right\}}_{i\in S_{k}},{\hat{M}}_{t}({\left\{y_{i}\right\}}_{i\in S_{k}}|S_{\text{train}})\right]$. The overall expected loss estimate is $\frac{1}{K}\sum_{k=1}^{K}l_{k}$. The notation for the expected loss over each test set is deliberately not summed over the indices of the testing data as this article considers non-additive loss functions, e.g. aggregate functions of the testing data. The choice of the number of folds *K* is context dependent and relates to a bias-variance trade-off: smaller *K* implies a smaller training set and thus increased positive bias in the error rate estimate, however, smaller *K* also forces greater dissimilarity between the training sets and thus lowers variance in the overall error estimate. The bootstrap is similar to 3-fold cross-validation, whereby approximately two-thirds of the data are used in the training set taken as a bootstrap sample of size *N*, constructed by bootstrapping (sampling with replacement). Predictive error estimation is then done by averaging the out-of-bag errors. The bootstrap generally improves on standard *K*-fold cross-validation as it smooths the predictive error when using discontinuous loss functions.

*K*-fold cross-validation and the bootstrap provide nearly unbiased estimators of the conditional expected loss if the empirical distribution $\hat{F_{x}}$ (in this context *x* denotes a molecule) is an i.i.d. draw from the true underlying data-generating distribution (Devroye *et al.*, 1996). In applications where the goal is to accurately predict the outcome of new data drawn at random with respect to a given data-generating process, these are the correct methods for selecting an optimal predictive model. However, drug (lead candidate) discovery is better thought of as a complex optimization problem rather than a passive data prediction problem. The goal here is to generalize (extrapolate) from a model trained on a relatively small dataset to find active molecules in a high-dimensional space ($2^{P}$ possibilities in total).

The data-generating distribution (e.g. the underlying processes which gave rise to the data at hand: this can be thought of as the experimental protocols which lead to the data-generating assays) will be substantially different from the uniform distribution over the subset of feasible molecules within the $2^{P}$ possibilities (Wallach and Heifets, 2018). This subset of feasible molecules is unknown and extra modelling procedures are needed to approximate it (Firth *et al.*, 2015). Validation methods based on random partitioning of available data give biased estimates of the true out-of-sample loss (Braga-Neto *et al.*, 2014; Wood *et al.*, 2007). For example, the data might be clustered together (with respect to Manhattan distance over the space of fingerprints) and therefore the out-of-sample estimate may in fact be highly skewed towards the in-sample estimate, leading to overconfidence. Therefore, it is necessary to partition the data in such a way that the out-of-sample testing subset is truly distinct from the in-sample data. We argue here that ‘distinct’ may not exactly map onto chemical dissimilarity measures but should be defined with respect to the outcome of interest. In this way, the partition should reflect the decision problem at hand and give reliable expected loss estimates which do not favour models that overfit to the training data. We next describe non-random data partitions which create ‘distinct’ training and testing sets motivated from a decision-theoretic perspective.

#### 3.1.2 Activity dependent model validation

In theory, it would be possible to determine whether a given training set is ‘close’ to a test set using a similarity metric on the molecular fingerprint space. In this context, ‘close’ is relative to the metric of choice. Metrics such as the Manhattan distance may be a poor proxy of this true (target specific) distance between subsets of data. Instead, we propose using the observed outcome (activity) *y* as the discriminant measure between molecules. Data partitions based on the activity function *G* (function relating the molecular fingerprint to the p-IC_50_) instead of random partitions force dis-similarities between subsets in the partition. If $G(x_{1})\gg G(x_{2})$, we assume that *x*_1_ is experimentally significantly different from *x*_2_.

The following validation design is proposed. Let $\hat{F_{y}}$ be the empirical distribution over the activities ${\left\{y_{i}\right\}}_{i=1}^{N}$. Let q∈(0,1) be a fixed fraction of the data used to determine the training set. With respect to the empirical distribution $\hat{F_{y}}$, this maps onto an activity threshold *Y_q_* (the *q*th quantile of $\hat{F_{y}}$). The training set is then constructed by bootstrapping the molecules with activity less than *Y_q_*. The testing set contains all the molecules with activity greater than *Y_q_*. This is the opposite of standard balanced or stratified cross-validation where one assures equal distributions of outcomes across the testing folds (Breiman *et al.*, 1984) and is not a ‘cross-validation’ design as the test data are never used as training data.

Multiple bootstrapped iterations are then computed in order to construct confidence intervals around the out-of-sample expected loss estimate. This can be thought of as a stabilizing process within the validation procedure (Efron, 1983).

In the following, we assume that the molecule index corresponds to the rank of the activities: $y_{1}\leq y_{2}\leq \ldots \leq y_{N}$. Let $N_{q}=\left\lfloor N\times q\right\rfloor$ be the number of elements in the training set based on the *q*th quantile.

For each model *M_t_*, evaluate for a=1,…,A independent iterations:

- Sample with replacement *N_q_* elements from ${\left\{x_{i}\right\}}_{i=1}^{N_{q}}$ to get a bootstrapped training dataset $X_{a}^{q}$.
- Compute $l_{a}=L\left[{\left\{y_{i}\right\}}_{i=N_{q}+1}^{N},M_{t}\left({\left\{y_{i}\right\}}_{i=N_{q}+1}^{N}|X_{a}^{q}\right)\right]$, where two proposals for the loss function *L* are given in the next section.

The set $\left\{l_{1},\ldots ,l_{A}\right\}$ is then used to estimate the mean expected loss, $\frac{1}{A}\sum_{a=1}^{A}l_{a}$, and the 95% confidence intervals.

#### 3.1.3 ‘Active-rank’ loss function

In the context of using statistical or machine learning methods for novel compound drug discovery, out-of-sample performance should not directly map onto standard goodness-of-fit measures (e.g. *R*^2^ or mean squared error), but has a simpler decision-theoretic interpretation. If these models are to be used in a real setting then a prediction of high activity for a given feature vector (fingerprint) would lead to a physical experiment confirming or refuting this prediction. As stated above, the goal is to find molecules with an activity above a certain threshold (this will be target specific) and therefore each bad prediction (whereby the true activity is less than the threshold) incurs a fixed loss (opportunity-cost and cost of experiment). In reality, experimental costs will not be constant (some molecules are more expensive to make than others); however, we simplify the situation to one where each experiment is considered to have a fixed cost. In the out-of-sample predictions, minimizing the loss corresponds to ranking the active molecules highest. When evaluating the performance of multiple models fitted to a given dataset, if there is one active molecule and a large number of inactive molecules, the expected loss is insensitive to the ranking of all the inactives below the rank of the active(s). The model’s accuracy within the region of the inactives is of no importance. This contrasts with standard measures of predictive accuracy and loss previously used in this context, such as *R*^2^, mean squared error or receiver operating characteristics (AUC) (Cumming *et al.*, 2013; Giguere *et al.*, 2013; Sheridan, 2013; Svetnik *et al.*, 2003; Wallach and Heifets, 2018; Wu *et al.*, 2018).

We define our ‘active-rank’ loss function as follows. We choose a quantile γ∈(0,1),γ>q, corresponding to a threshold activity $Y_{\gamma }$ with respect to the empirical distribution function $\hat{F_{Y}}$. In practice, *γ* would be close to 1 (e.g. in the range 0.9–0.99) to simulate scenarios where actives molecules are rare and inactives common. The subset of molecules ${\left\{x_{i}\right\}}_{i=N-N_{\gamma }+1}^{N}$ are then defined as ‘actives’. We define $N_{\gamma }=\left\lfloor N\times (1-\gamma )\right\rfloor$ (the total number of actives), and $N_{\text{test}}=N-N_{q}$ (the size of the test set).

For the model ${\hat{M}}_{t}$ fit to the training data ${\left\{x_{i}\right\}}_{i=1}^{N_{q}}$, the out-of-sample loss is defined with respect to the ranks assigned to the out-of-sample active molecules. We take as convention that the ranks assigned to the test data go from 0 (molecule with highest predicted activity) to $N_{\text{test}}-1$ (molecule with least predicted activity). The loss which only depends on the rank of the highest ranked active is defined as:

(1) $$L_{\min}^{\gamma }=\frac{1}{N_{\mathrm{test}}-N_{\gamma }}\underset{j=N-N_{\gamma }+1,\ldots ,N}{\min}{\text{Rank}}_{M_{t}}(x_{j})$$

The minimum active rank will vary from 0 (an active molecule is ranked top in the test data), to $N_{\text{test}}-N_{\gamma }$ (all the $N_{\gamma }$ active molecules are ranked last). We normalize to obtain a loss function defined over the interval [0, 1]. An alternative version of this loss, whereby all the ranks of the active molecules are taken into account, thus penalizing sub-optimal ranking for all active molecules, is given by:

(2) $$L_{\text{sum}}^{\gamma }=\frac{\sum_{j=N-N_{\gamma }+1}^{N}{\text{Rank}}_{M_{t}}(x_{j})-N_{\gamma }(N_{\gamma }-1)/2}{N_{\gamma }(N_{\mathrm{test}}-N_{\gamma }-1)}$$

The sum of the active ranks will vary from $N_{\gamma }(N_{\gamma }-1)/2$ (all actives are ranked in the top $N_{\gamma }$ molecules) to $N_{\gamma }(2N_{\text{test}}-N_{\gamma }-1)/2$ (all actives are ranked last).

We note that when $N_{\gamma }=1$, e.g. there is only one active molecule, $L_{\text{min}}^{\gamma }=L_{\text{sum}}^{\gamma }$.

As mentioned above, both these loss functions are non-additive with respect to the testing data.

#### 3.1.4 Assessing similarity of molecules

In order to characterize better how splitting by activity corresponds to selecting molecules that are more or less ‘similar’ to each other, we assess similarity within training and testing sets using the Tanimoto distance (also known as the Jaccard metric). Under our notation, this is defined as:

(3) $$D(x_{1},x_{2})=\frac{\sum_{j=1}^{P}x_{1}^{j}x_{2}^{j}}{\sum_{j=1}^{P}\max(x_{1}^{j},x_{2}^{j})}$$

This is the number of substructures shared between *x*_1_ and *x*_2_ over all the substructures present in either one of the molecules.

### 3.2 Statistical analysis

All statistical analyses were done in Python version 2.7. The entire analysis is fully reproducible via a publicly available Python Jupyter notebook found at https://github.com/owatson/QuantileBootstrap.

#### 3.2.1 Regression models

We evaluated the performance of four model classes:

- Support vector regression (Python module: *sklearn*, function *SVR*)
- Random forests (Python module: *sklearn*, function *RandomForestRegressor*)
- Linear ridge regression (Python module: *sklearn*, function *Ridge*)
- Deep neural networks (Python module: *sklearn*, functions *Pipline* and *StandardScalar*, and Python module: *keras*, function *KerasRegressor*).

For support vector regression, we used a linear kernel. For random forests, we used the default parameter settings, growing 100 trees each with a maximum tree depth of 10 splits. For linear ridge regression, we used a penalty term of α=0.1. For deep neural networks, we first standardized the data, then used two dense layers, the first of dimension 128 (to match the input feature dimension) and then dimension 16, both with relu activation.

These correspond to standard default choices in the literature. These four model classes are all somewhat insensitive to tuning parameters. In order to minimize any optimization bias, we did not attempt to tune any of the parameters to the set of datasets at hand.

#### 3.2.2 Model comparison

We first compared model performances using 5-fold cross-validation (this uses 80% of data chosen at random to predict the remaining 20%) and bootstrapping (this uses approximately two-thirds of the data to predict the remaining third). With discontinuous loss functions, bootstrapping smooths the out-of-sample error predictions Efron (1983). The out-of-sample predictions we evaluated using mean squared error, and both active-rank loss functions $L_{\text{min}}^{\gamma }$ and $L_{\text{sum}}^{\gamma }$. For the active-rank loss functions, we evaluated out-of-sample loss using three separate *γ* thresholds corresponding, respectively, to labelling 10%, 5% and 1% of the test data as active.

We then ran our activity dependent validation procedure using progressively lower fractional thresholds for the training data: q=0.9;0.8;0.6;0.4. The same three *γ* thresholds were used to evaluate the out-of-sample expected losses for the active-rank loss functions. All predictions were evaluated with mean squared error and both active-rank loss functions.

Overall performance was evaluated by assuming independence between the 25 datasets. The total model score assigned to each model *M_t_* is defined as the sum over all datasets of the probabilities that the *M_t_* had lowest expected loss (probability of optimality). As the number of bootstrap iterations is much lower than the total number of possible iterations ($N_{q}^{N_{q}}$), we use the jackknife to calculate the standard error on the mean out-of-sample prediction Efron (1983). 400 bootstrap iterations were used for each model and set of problem definition parameters, i.e. the pair of parameters (q,γ).

### 3.3 Data

#### 3.3.1 Data curation

We extracted IC_50_ data from ChEMBL database version 23 for 25 diverse protein targets and receptors. In order to assemble high-quality datasets, we only considered IC_50_ values for compounds that satisfied the following filtering criteria: (i) an activity unit equal to ‘nM’, (ii) activity relationship equal to ‘=’, (iii) target type equal to ‘SINGLE PROTEIN’ and (iv) organism equal to *Homo sapiens*. Bioactivity values were modelled in a logarithmic scale (i.e. pIC_50_$=-{\;\text{log}\;}_{10}{\text{IC}}_{50}$). The average pIC_50_ value was calculated for protein-compound pairs with multiple IC_50_ measurements available.

Further details about the datasets are provided in Table 1. A comparative analysis of these datasets was performed previously in the context of iterative model fitting (Cortes-Ciriano *et al.*, 2018). All data used in this article (activity levels, 128-bit fingerprints and smiles) are available at: https://github.com/owatson/QuantileBootstrap.

**Table 1.** Twenty-five publicly available datasets extracted from ChEMBL and analysed in this article

Target preferred name	Target abbreviation	Uniprot ID	ChEMBL ID	#Bioactive molecules
Alpha-2a adrenergic receptor	A2a	P08913	1867	203
Tyrosine-protein kinase ABL	ABL1	P00519	1862	773
Acetylcholinesterase	Acetylcholin	P22303	220	3159
Androgen Receptor	Androgen	P10275	1871	1290
Serine/threonine-protein kinase Aurora-A	Aurora-A	O14965	4722	2125
Serine/threonine-protein kinase B-raf	B-raf	P15056	5145	1730
Cannabinoid CB1 receptor	Cannabinoid	P21554	218	1116
Carbonic anhydrase II	Carbonic	P00918	205	603
Caspase-3	Caspase	P42574	2334	1606
Thrombin	Coagulation	P00734	204	1700
Cyclooxygenase-1	COX-1	P23219	221	1343
Cyclooxygenase-2	COX-2	P35354	230	2855
Dihydrofolate reductase	Dihydrofolate	P00374	202	584
Dopamine D2 receptor	Dopamine	P14416	217	479
Norepinephrine transporter	Ephrin	P23975	222	1740
Epidermal growth factor receptor erbB1	erbB1	P00533	203	4 868
Estrogen receptor alpha	Estrogen	P03372	206	1705
Glucocorticoid receptor	Glucocorticoid	P04150	2034	1447
Glycogen synthase kinase-3 beta	Glycogen	P49841	262	1757
HERG	HERG	Q12809	240	5207
Tyrosine-protein kinase JAK2	JAK2	O60674	2971	2655
Tyrosine-protein kinase LCK	LCK	P06239	258	1352
Monoamine oxidase A	Monoamine	P21397	1951	1379
Mu opioid receptor	Opioid	P35372	233	840
Vanilloid receptor	Vanilloid	Q8NER1	4794	1923

#### 3.3.2 Molecular representation

The python module *Standardizer* was used to standardize all chemical structures. Inorganic molecules were removed, and the largest fragment was kept in order to filter out counterions.

We computed circular Morgan fingerprints 52 using RDkit (release version 2013.03.02). The radius was set to 2 and the fingerprint length to 128.

## 4 Results

### 4.1 Model performance evaluated using random data partitioning

**Fig. 1.**
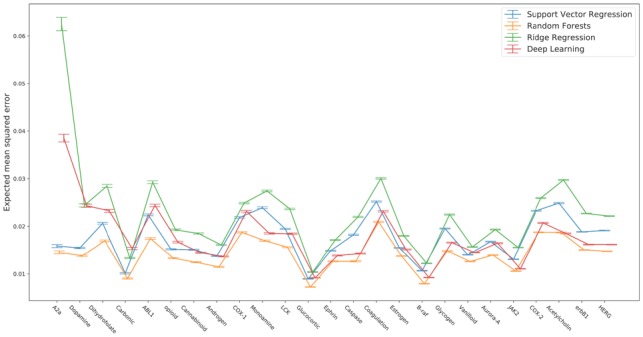
Model comparison using the standard bootstrap. Expected model out-of-sample mean squared error shown for each dataset, ordered from left to right by increasing size of dataset. Error bars correspond to ±2 standard errors around the expected loss estimate, computed using the jackknife estimator. For each dataset, the optimal model is the one with least expected loss, with random forests scoring best for every single dataset. Datasets are ordered from left to right by increasing size

Random partitioning of the data, either using 5-fold cross-validation (training set contains 80% of the data) or bootstrapping (training set contains two-thirds of the data) resulted in random forests and deep learning having the best out-of-sample performance (e.g. Fig. 1; for full results see github Jupyter noteboook). Figure 1 shows the bootstrap out-of-bag performance as evaluated by mean squared error for the four models over the 25 datasets, with datasets ordered from smallest to largest. Ridge regression, in the majority of cases, has the largest out-of-bag error, followed by support vector regression and then deep-learning and random forests.

**Fig. 2.**
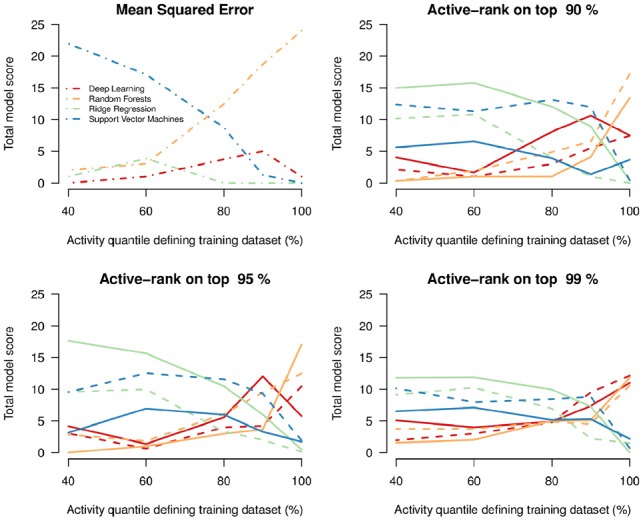
Comparison of overall model performance for the standard bootstrap and the restricted activity bootstrap. All four panels show the overall model score (sum of the probabilities of model optimality over the 25 datasets) as a function of the restriction on the activity levels in the training data. About 100% corresponds to standard cross-validation (random partitioning). The first three panels show the results for the active-rank loss functions ($L_{\min^{\gamma }}$ shown by thick lines; $L_{\text{sum}}^{\gamma }$ shown by dashed lines) with values of *γ* going from 0.9 (top left) to 0.99 (bottom left). The bottom right panel shows the results when models are scored using mean squared error (dot-dashed lines). Red: deep learning; blue: support vector regression; orange: random forests; green: ridge regression

Overall model performance using random data partitioning is shown in Figure 2, corresponding to the point on the *x*-axis at 100%. When scored using mean squared error (Fig. 2, top left panel), and performs on average as well as deep learning when scored with the active-rank loss functions (Fig. 2, last three panels). Ridge regression and support vector regression are never optimal in this setting, irrespective of the loss function.

These out-of-sample performances closely reflect the in-sample error. Both deep learning and random forests can almost ‘memorize’ the data with in-sample losses close to zero (see Jupyter notebook).

### 4.2 Quantile bootstrap

Decreasing the quantile-activity threshold for the training data from 1 (random partitioning described above) to 0.4 (only 40% of the data ordered by activity are used in the bootstrap construction of the training set) results in a complete reversal of optimality amongst the four predictive models. When scoring models by out-of-sample mean squared error, support vector regression becomes optimal for quantiles below 0.75 (Fig. 2, bottom right panel).

**Fig. 3.**
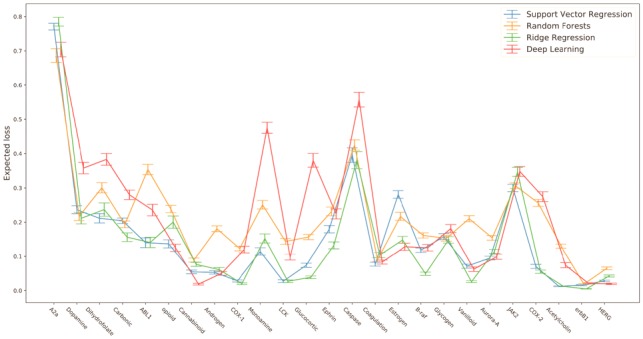
Model comparison using the restricted activity bootstrap with γ=0.4. Model expected out-of-sample $L_{\text{min}}^{\gamma =0.99}$ loss shown for each dataset, ordered from left to right by increasing size of dataset. Error bars correspond to ±2 standard errors around the expected loss estimate, computed using the jackknife estimator. For each dataset, the optimal model is the one with least expected loss. Datasets are ordered from left to right by increasing size

For the active-rank loss functions, lowering the activity training threshold also induces a reversal of model optimality (change-point for q≈0.8). In the most extreme setting (*q *=* *0.4), support vector regression and ridge regression perform approximately equally well, with total scores corresponding to optimality on half of the datasets (shown in detail in Fig. 3). There is some heterogeneity between the datasets for model optimality, but the overall trends are clearly in favour of both ridge regression and support vector regression.

By averaging over the 25 datasets, we can see that these trends are robust with respective the target used as the outcome measure in the regression models.

### 4.3 Comparison with similarity-based unsupervised clustering

We explored whether unsupervised clustering could be used to construct training and testing sets that maximize similarity within clusters and dissimilarity across clusters. For this, we used a two-mediods clustering algorithm (Park and Jun, 2009) with similarity defined by the Tanimoto distance metric. For the 25 datasets presented here, unsupervised clustering did not achieve good reductions in dissimilarity: the average pairwise distance within each cluster was only approximately 2% lower than the global average pairwise distance (see Supplementary Materials). In addition, when we compared the results to using an activity dependent split of the data (e.g. at the 90th quantile of activity) this achieved greater reductions in dissimilarity within the testing set (the molecules of high activity). In summary, this empirically shows that it is difficult to cluster molecular data based on a similarity metric. However, in many of the datasets analysed here, the high-activity molecules are highly similar to one another. This is likely due to bias in the experimental workflows. This reinforces the use of activity splitting to assess model extrapolation performance.

### 4.4 Importance of the loss function

There are clear disparities between model evaluations for the different loss functions. Mean squared error favours random forests in the standard setting (*q *=* *1), and support vector regression in the restricted activity setting (*q *<* *0.6). However, the active-rank loss functions favour equally deep-learning and random forests in the standard setting, and support vector regression and ridge regression in the restricted activity setting. Moreover, the results differ between $L_{\text{min}}^{\gamma }$ and $L_{\text{sum}}^{\gamma }$. The out-of-sample performance of ridge regression is consistently better when evaluated using $L_{\text{min}}^{\gamma }$, and that of support vector regression is consistently better when evaluated using $L_{\text{sum}}^{\gamma }$ (Fig. 2). These results show that the evaluation of model performance is highly dependent on the loss function used. This directly reflects how the different loss functions penalize predictive performance, with $L_{\text{min}}^{\gamma }$ only penalizing the rank of the first active molecule. We note that in this setting, for the 25 datasets, the four model families and three loss functions used here, the sample size of the training sets does not affect the overall relative performance on the testing sets (Supplementary Figure S2).

We also note that using mean squared error to evaluate the performance of random forests unfairly penalizes the model fit. For quantile bootstraps with *q *<* *1, random forests cannot predict activities greater than the maximum activity in the training set. Therefore the contribution to the mean squared error from high-activity molecules will all be from bias rather than variance in the prediction (predictions will be systematically lower). This is in contrast to using rank-based loss functions which do not suffer from this bias issue.

From a subjective Bayesian perspective (Savage, 1954), the choice of loss function reflects the decision task at hand. This should be specified separately from the regression model. The two active-rank loss functions are examples of possible choices of loss functions. However, other, more standard choices, are also possible. For example, the Spearman rank correlation coefficient, or the F beta score on thresholded predictions. It is important to note that assessment of models may be sensitive to the choice of loss and careful consideration of the decision goals is needed.

## 5 Discussion

There is considerable hype around the use of artificial intelligence and machine learning to find novel drug candidates and to optimize early-stage drug discovery (Fleming, 2018). Deep learning via the use of deep neural networks is a highly active research area with a wide range of applications and proven success stories. However, neural networks are known to be extremely ‘data-hungry’ and work best in high signal-to-noise settings (Marcus, 2018). For regression modelling using molecular structure-activity data, we do not believe deep-learning models will perform well in predicting novel areas of molecular space of high activity, contrary to recent claims (Lenselink *et al.*, 2017). This modelling exercise empirically shows that partitioning on quantiles of the activity distribution, and thereby mimicking the process of extrapolating onto previously unseen areas of molecular space, removes the predictive advantage from the deep-learning models. This approach can be contrasted with ‘temporal splitting’ whereby datasets are partitioned by assay date, the first section used to train the model, the second to test. Temporal splitting is easy to understand and could be argued to mimic real-life settings, but it does not provide any rigorous guarantees. It does not guarantee that highly similar molecules—both in structure and activity—will not be found across both testing and training data. Time of assay will not always correspond to time of conception and therefore ‘worse’ molecules could have been tested at later dates. Drug discovery does not follow a linear process nor does it directly test the capability of a statistical or machine learning model to detect signal predicting activity gradients, resulting in good predictions of molecules with high activity. Splitting on activity quantiles deals with these issues, and provides a simple and interpretable univariate parametrization of the information content used to train the model. We note that a methodological limitation of the quantile-activity bootstrap method is the inability for the regression algorithm to learn about potential ‘activity cliffs’. If there was a large activity cliff, then the low-activity molecules would be in the training data, and the proximal high-activity molecules in the testing data. However, the impact of the limitation is dependent on the decision task at hand. If the overall goal is to assess the extrapolation properties of a model then there is ‘no free lunch’: it is necessary to put aside data for testing and these data cannot also be used for training.

Evaluation of the predictive performance of regression models when applied to small-molecule structure-activity datasets necessitates different approaches than in the standard bioinformatic and high-dimensional settings. Online prediction problems (e.g. image classification, spam filtering, recommender systems, etc.) and statistical inference problems (e.g. genome-wide studies, biomarker discovery, micro-array analysis) have different goals. In the drug discovery context, we start with a small training set ($N\ll 2^{P}$) and attempt to extrapolate outside of these data in order to find molecules which are inherently ‘different’ from those in the training data. In the machine learning and computational statistics literature, this is most similar to an optimization problem or gradient ascent problem. This search procedure is done in a relatively resource constrained setting (cost of experimentation, time cost) and therefore model evaluation should be decision theoretic with a subjective loss (Savage, 1954).

We expect our active-rank loss functions to differ in performance from standard machine learning type losses (most commonly this would be mean squared error). The active-rank loss functions $L_{\text{min}}^{\gamma }$ and $L_{\text{sum}}^{\gamma }$ do not penalize bad predictions outside of the subspace of interest, i.e. high-activity areas of molecular space. In addition, these loss functions are non-additive and therefore one limitation is that they cannot be used to penalize model fitting in the training phase. However, the use of non-additive loss functions fits our proposed conceptual workflow for computational drug discovery (Cortes-Ciriano *et al.*, 2018). In the first stage, existing software such as Firth *et al.* (2015) can be used to construct sets of viable molecules similar to existing molecules with reasonable potency. In the second stage, computational algorithms are then trained to existing structure-activity datasets. Finally, fitted models are then used to rank molecules constructed in stage 1 and the highest ranked can then be tested *in vitro*. Other limitations of the work are that we have done little to no internal model parameter tuning, except for deep neural nets to assess structures most appropriate for these types of data. However, we do not expect parameter tuning to considerably change the results nor the conclusions of the study. Furthermore, all the analyses are easily reproducible with our openly available Jupyter notebook, thus easily extended to new computational algorithms, different parameter settings or new datasets. Lastly, the loss functions used to evaluate model performance on these benchmark datasets will not estimate the true out-of-sample expected loss in experimental settings. In reality, true *γ* thresholds (percentage of feasible molecules above a certain activity level) could be multiple orders of magnitude larger than those used in our study (e.g. the top $10^{-10}$% of the testing data).

*Conflict of Interest*: For ART and JAW. OPW and ICC have shares in Evariste Technologies.

## Supplementary Material

btz293_Supplementary_Materials
