# Supplementary material for: A decision-theoretic approach to the evaluation of machine learning algorithms in computational drug discovery
Source: Bioinformatics. 2019 May 9;35(22):4656–63. doi: 10.1093/bioinformatics/btz293 (PMC6853675; doi:10.1093/bioinformatics/btz293)
Supplement: btz293_Supplementary_Materials [file btz293_supplementary_materials.pdf]

Supplementary materials: A decision-theoretic approach to the evaluation of machine learning algorithms in computational drug discovery

Oliver P Watson, Isidro Cortes-Ciriano, Aimee R Taylor, James A Watson

March 22, 2019

S1 Similarity clustering

We compared the construction of training and testing sets using an unsupervised clustering algorithm to using activity-based quantile splits. In the activity-based quantile split we define cluster 1 as all molecules with activity below the qth activity quantile, and cluster 2 as all the molecules with activity above the qth activity quantile.

Figure S1 shows for each dataset (ordered from left to right by sample size) the average intra-cluster pairwise distance (using the Tanimoto distance) relative to the global average pairwise distance. We note that there is little gain in terms of similarity for the clusters defined using unsupervised 2-mediods clustering. For the quantile-activity based clustering, the cluster 1s (low activity) have slightly greater diversity relative to the entire datasets (values greater than 1). However the cluster 2s have lower diversity, especially in some of the datasets, notable A2a and Aurora-A. This suggests that the high activity molecules in these datasets are highly similar to each other.

S2 Sensitivity of regression algorithms to sample size

To assess the sensitivity of each regression model to the sample size of the training set, we ran the same analysis but after randomly permuting the ranks (no longer activity based splitting). Figure S2 shows the relationship between the estimated loss (over all datasets) and the sample size used for bootstrapping. There is very little effect with no overall trends for any of the models.

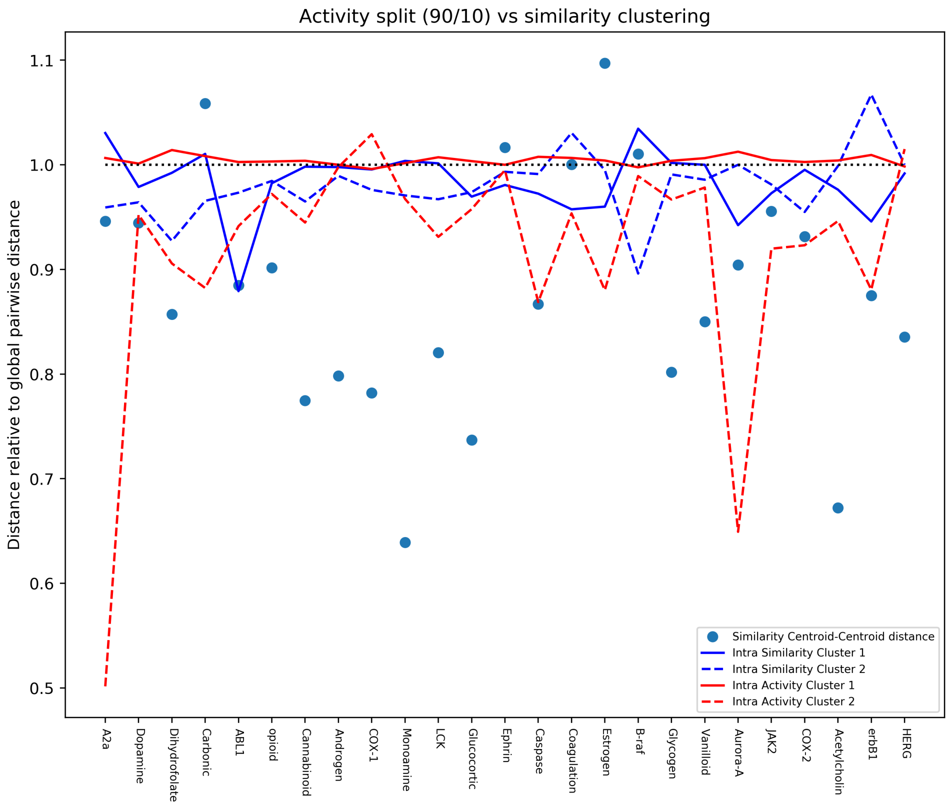

Figure S1: Comparison of average pairwise intra-cluster distances (Tanimoto distance) using a 2-Mediods clustering algorithm (blue) and using activity based clusters (red). The distances are shown as relative to the global average pairwise distance between molecules in each dataset. The blue dots show the relative distances between centroids of each similarity based cluster.

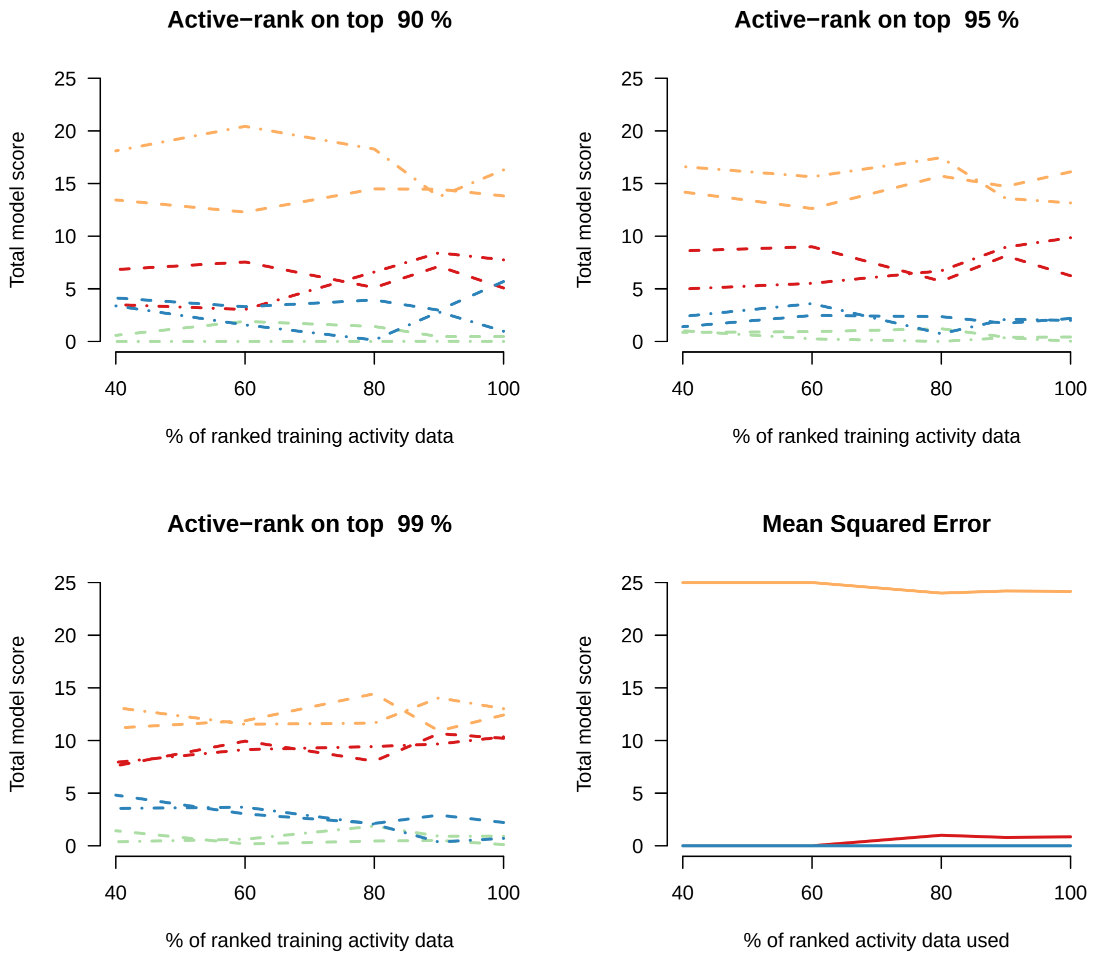

Figure S2: Relationship between training set sample size and the out-of-sample loss.
